# Data on characterization and validation of assays for ultrasensitive quantitative detection of small molecules: Determination of free thyroxine with magnetic and interferometric methods

**DOI:** 10.1016/j.dib.2018.10.145

**Published:** 2018-11-01

**Authors:** A.V. Orlov, S.L. Znoyko, A.V. Pushkarev, E.N. Mochalova, N.V. Guteneva, A.V. Lunin, M.P. Nikitin, P.I. Nikitin

**Affiliations:** a Prokhorov General Physics Institute of the Russian Academy of Sciences, 38 Vavilov St., Moscow 119991, Russia; b Moscow Institute of Physics and Technology, 9 Institutskiy per., Dolgoprudny, Moscow Region 141700, Russia; c National Research Nuclear University MEPhI (Moscow Engineering Physics Institute), 31 Kashirskoe shosse, Moscow 115409, Russia

## Abstract

The presented data refer to optimization and quantitative characterization of a rapid lateral flow assay based on high-affinity bifunctional ligand and magnetic nanolabels, which was developed for detection of small molecules of thyroid hormones. The results were obtained by several techniques, including the magnetic particle quantification method, spectral-correlation interferometry and spectral-phase interferometry, dynamic light scattering, enzyme linked immunosorbent assay. The long-term stability of “antibody – magnetic nanoparticle” conjugates is shown. The assay specificity is confirmed, and verification of successful combination of magnetic particles and antibodies is demonstrated. The kinetic and equilibrium dissociation constants are determined for interactions between thyroxine and monoclonal antibodies. The obtained data could be used for design of other platforms for detection of small molecules.

**Specifications table**

**Table:** 

Subject area	Analytical chemistry
More specific subject area	Detection of small molecules in complex media
Type of data	Figures
How data were acquired	Magnetic particle quantification (MPQ) method; spectral-correlation interferometry (SCI); spectral-phase interferometry (SPI); dynamic light scattering (DLS); enzyme linked immunosorbent assay (ELISA)
Data format	Analyzed
Experimental factors	2 µl of the magnetic particles conjugated with antibody was added to the calibration samples of human blood serum (80 µl volume) with different concentrations of free thyroxine, mixed by vortex and incubated for 5 min. Then the biotinylated thyroxine was added and mixed by vortex. Lateral flow test strip with streptavidin on a test line was placed vertically into the samples. To detect magnetic signal, a test strip was inserted into a measuring coil of the MPQ reader. The spectral-phase interferometry and spectral-correlation interferometry were used to determine the kinetic parameters of binding of immumoreadents and conjugated magnetic particles.
Experimental features	The magnetic nanolabels were counted by original MPQ-readers using inductive registration of non-linear magnetic materials at combinatorial frequencies. The label-free biosensors were used for evaluation of kinetic performance. The devices use microscopic cover glass slips as the sensor chips and provide real-time monitoring of changes in thickness of a biomolecular layer bound on the sensor chip surface, averaged over the sensing area.
Data source location	Moscow, Russia
Data accessibility	Data are presented in this article
Related research article	S.L. Znoyko, A.V. Orlov, A.V. Pushkarev, E.N. Mochalova, N.V. Guteneva, A.V. Lunin, M.P. Nikitin, P.I. Nikitin, Ultrasensitive quantitative detection of small molecules with rapid lateral-flow assay based on high-affinity bifunctional ligand and magnetic nanolabels, Anal. Chim. Acta. 1034 (2018) 161–167. [1]

**Value of the data**

● The data can be used for design and validation of other platforms for ultrasensitive detection of small molecules.
● The characterization and quantitative optimization of all stages of magnetic lateral flow assay were carried out, including characterization of the obtained conjugates, their specificity and sensitivity, validation of long-term stability of “antibody – magnetic nanoparticle” conjugates and determination of dissociation constants for thyroxine and anti-thyroxine antibodies.
● The described methods and results are important for simple and rapid design of assays without significant reagent consumption.

## 1 Introduction

The obtained data show optimization and validation of all stages of the developed magnetic lateral flow assay for quantitative and ultrasensitive express-detection of free thyroxine [1]. The assay was performed in an original competitive format with streptavidin on the test line (TL) of a lateral flow test strip and biotinylated thyroxin (T4-bt) competing with free thyroxin for the limited amount of antibody binding sites in sample solution. The antibodies were conjugated with magnetic nanoparticles (MP) by carbodiimide method described in [2] and stored at +4 °C before use. The obtained conjugates (volume 2 μl) and 12 μl of T4-bt were premixed with 80–μl samples of human serum containing known concentrations of free thyroxin and incubated during 15 min at room temperature. After that, the lateral flow test strips with streptavidin on TL were placed vertically into the samples. The complexes “MP – antibody – T4-bt” bound to streptavidin on the test line and the complexes “MP – antibody – free T4” accumulated on the absorbent sink of lateral flow test strips. The signal from test line was measured by the Magnetic Particle Quantification (MPQ) readers described in details in [2], [3]. The original design and quantitative optimization of each stage of the assay has permitted attractive limit of detection for free thyroxin in human serum of 20 fM or 16 fg/ml. The high sensitivity of proposed assay is due to high-affinity “biotin-streptavidin” interactions and sensitive quantification of magnetic nanolabels from entire volume of the test zone on 3D strip membranes. The data below refer to assay characterization and can be used for effective design of rapid and sensitive test-systems for detection of small molecules.

## 2 Stability of “antibody – magnetic nanoparticle” conjugates

**Fig. 1:**
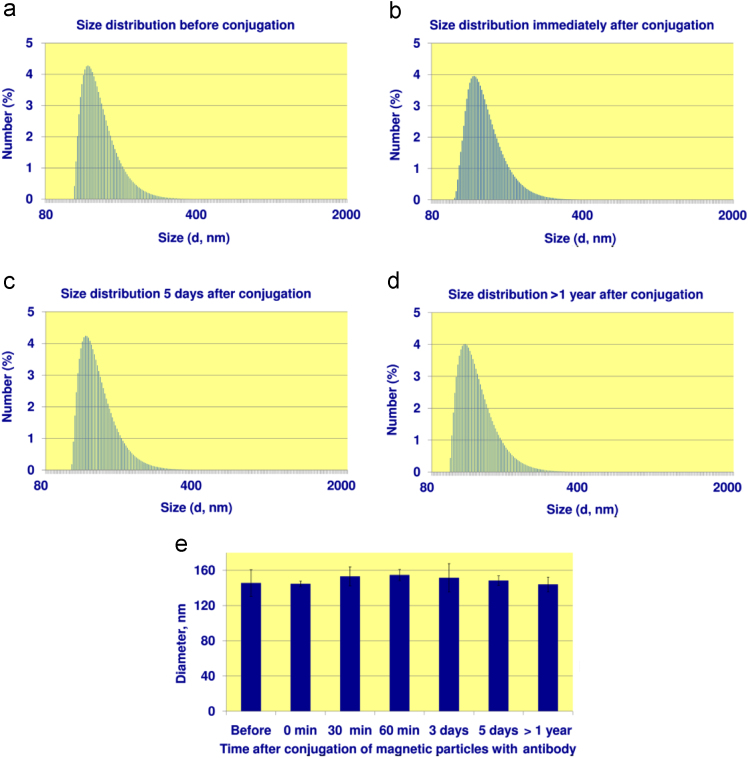
Size distribution measured by the dynamic light scattering. (a–d) Distribution for magnetic particles: before conjugation (a); immediately after conjugation with antibody to free thyroxine (b); after 5 days (c) and after a year (d) since conjugation. (e) Temporal evolution of mean diameter of the particles averaged for three independently conjugated samples. Error bars show standard deviation.

The obtained conjugates demonstrate high stability; they do not agglomerate and provide excellent performance in the assay for more than one year after conjugation. These facts were confirmed by dependence of size distribution of the conjugates on time lapse since conjugation. The dependences were obtained by the dynamic light scattering using (i) the magnetic particles before conjugation; (ii) the magnetic particles immediately after conjugation with antibody against free thyroxine; (iii) the particles that were conjugated 30 min, 60 min, 3 days, 5 days ago; and the conjugates prepared for this research more than a year ago and were stored at +4 °C in dH_2_O. The typical size distributions are shown in Fig. 1a–d. The temporal dependence of average diameter of the particles can be found in Fig. 1e. One can see that neither the distribution pattern, nor average diameter depend on time lapse since conjugation. No particle agglomeration occurs because the fraction of larger particles, as well as average diameter of the conjugates do not increase with time. In addition, each of these conjugates was used in the lateral flow immunochromatographic format. The specific signal at the test line was virtually independent on time lapse since conjugation.

## 3 Verification of successful combination of MP and antibody

To verify the successful combination of MP and antibody, we experimentally estimated the ratio of amount of antibody sorbed on MP surface to the amount of antibody added to the particles during conjugation, as well as the fraction of the sorbed antibody that retained antigen-binding activity. It was found that 70% of antibody introduced during conjugation bound with magnetic particles, among which 34% exhibited antigen activity.

**Fig. 2:**
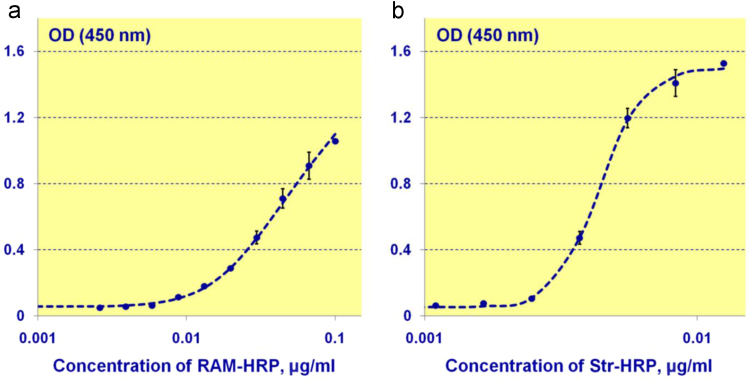
Calibration curves for determining the concentration of antibody bound with MP during conjugation (a) and concentration of antibody retaining activity after conjugation with MP (b).

To determine the concentration of antibody bound with MP during conjugation, anti-mouse antibody labelled with horseradish peroxidase was added to the obtained magnetic conjugates. After that, the non-bound antibody was removed by magnetic separation. The concentration of assembled antigen-antibody complexes was calculated using the calibration curve shown in Fig. 2а. For the calibration, we used solutions of labeled anti-mouse antibody in different concentrations. To plot the calibration curve, 5-parameter logistic curve was used.

The concentration of antibody remaining active on MP was determined using successive addition to the particles of biotinylated T4 and streptavidin labelled by horseradish peroxidase. The amount of formed complexes was found by absorbance at 450 nm and permitted one to infer the amount of active antibodies on MP surface. In that case, the calibration was implemented similarly (Fig. 2b) but solutions of labelled streptavidin were used instead of those of anti-mouse antibody.

## 4 Optimization of assay parameters

### 4.1 Antibody amount on magnetic nanoparticles

**Fig. 3:**
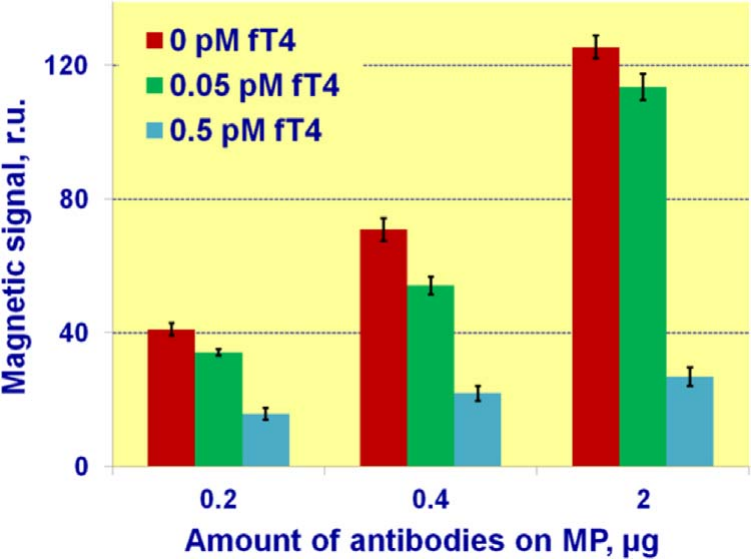
Dependence of magnetic signal on the test lines of lateral flow test strips upon antibody amount per 300 µg of magnetic nanoparticles for different concentrations of fT4.

For optimization of the antibody amount added to magnetic particles during conjugation, the magnetic signals on the TL were measured for three samples of human blood serum having different concentrations of free thyroxine (fT4) (0, 0.05 and 0.5 pM) at three different concentrations of Ab-MP conjugates in the sample (Fig. 3). The conjugates were obtained by adding different amounts of antibody (0.2, 0.4, and 2 μg) to the fixed amount of 300 μg of magnetic nanolabels. According to the experiments, the optimal ratio of the signal registered for blank serum (0 pM of fT4) to that recorded for the serum having small concentration of fT4 (0.05 pM) was achieved with 0.4 μg of Ab in the MP-Ab conjugate as shown in Fig. 3.

### 4.2 Concentration of magnetic conjugate in the sample

**Fig. 4:**
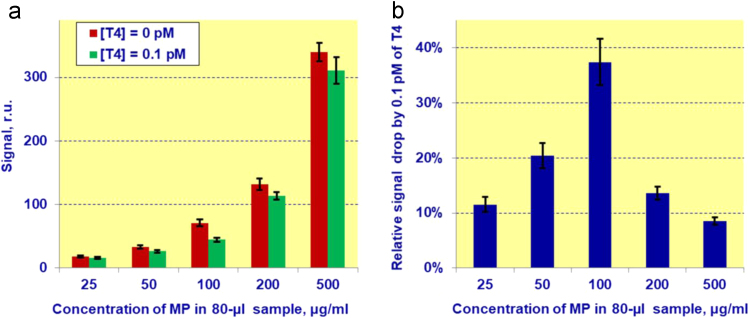
Dependences of magnetic signal on the test lines of lateral flow test strips (a) and relative signal decrease caused by 0.1 pM of free thyroxine upon concentration of magnetic conjugate in 80–µl samples.

The concentration of magnetic particles in samples of human blood serum with known amount of fT4, independently determined by supplier, was optimized to achieve the most pronounced decrease of the specific signal at the test line due to the presence in sample of low concentrations of fT4. For this purpose, the assay was carried out using negative control in the absence of thyroxine and positive control containing 0.1 pM of fT4 (Fig. 4a). It was found that the maximal signal reduction was recorded at 100 µg/ml of magnetic particles in 80-µl sample (Fig. 4b).

## 5 Specificity of the assay

**Fig. 5:**
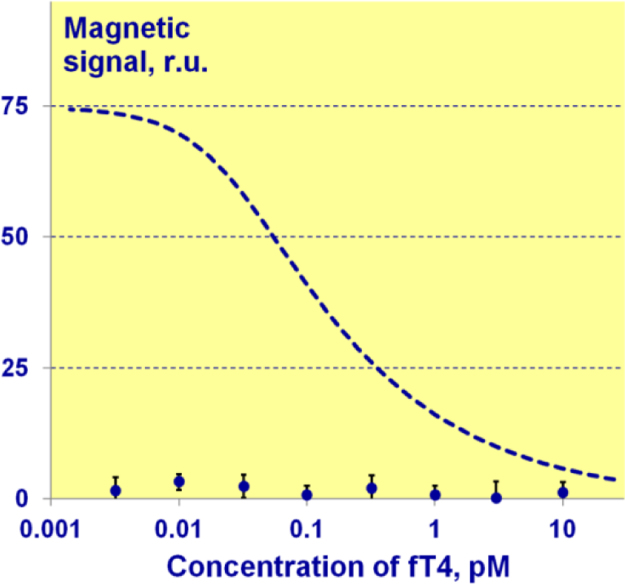
Dependence of the registered magnetic signal upon antigen concentration in the presence (dash line – see details in Fig. 4b in [1]) and absence (dots) of T4-bt conjugate in the sample.

To verify the specificity of competitive assay, the calibration curves on fT4 antigen concentration in the analyzed samples in the presence and absence of T4-bt were compared (Fig. 5). In the latter case, only magnetic particles conjugated with antibody were added to the serum containing different concentration of fT4. As no specific streptavidin/T4-bt/MP-Ab complexes were formed in these experiments, the magnetic signal on TL was due to non-specific interactions. The recorded signals remained constant for different concentrations of detectible antigen and did not exceed 3% of maximum magnetic signal value. Such small variations did not affect the calibration curve shape and limit of detection of the assay.

**Fig. 6:**
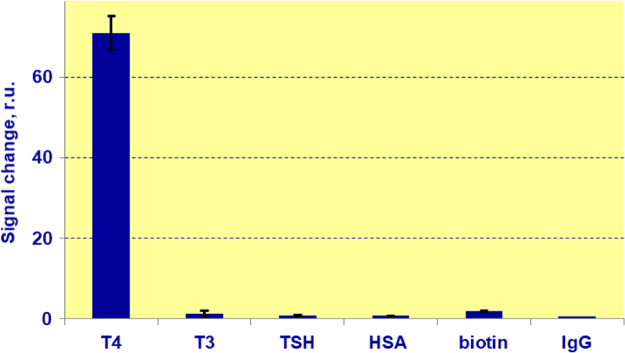
Changes in signal of the developed assay due to the presence of free thyroxine and non-target analytes in samples.

The specificity of the developed assay is also confirmed by the fact that presence of physiologic concentrations of other non-target molecules such as triiodothyronine (T3), thyroid-stimulating hormone (TSH), human serum albumin (HSA), biotin, human immunoglobulin G also has no effect on the assay results (Fig. 6).

## 6 Determination of the equilibrium dissociation constant of thyroxine interaction with monoclonal anti-thyroxine antibody

**Fig. 7:**
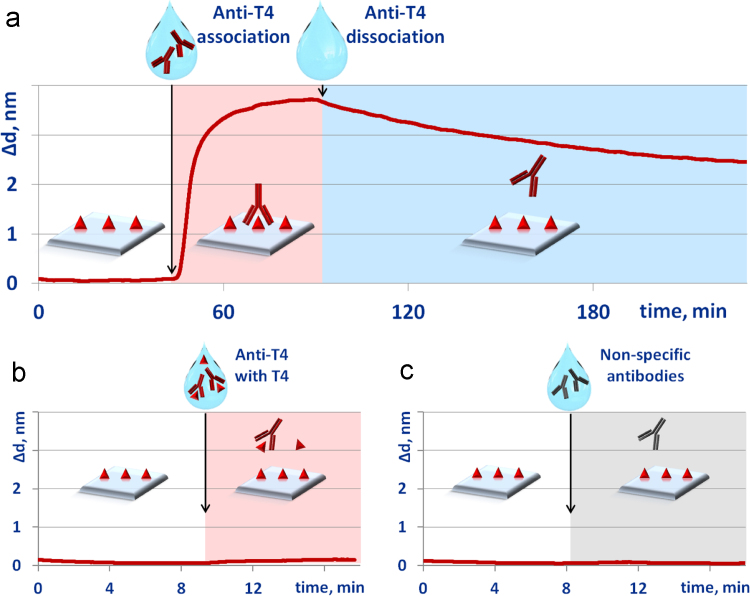
Characteristic sensograms of interactions of thyroxine on a sensor chip surface with different reagents: (a) sorption-desorption of monoclonal antibody against thyroxine; (b) interaction with monoclonal antibody against thyroxine in the presence of free thyroxine; (c) interaction with non-specific antibody.

The equilibrium dissociation constant of thyroxine interaction with monoclonal anti-thyroxine antibody was determined. To monitor antibody association, solution of 100 μg/ml anti-thyroxine antibody in phosphate buffered saline (PBS) (pH = 7.4) was passed along the sensor chip with L-thyroxine immobilized on its surface. Upon reaching the adsorption-desorption equilibrium (sensogram plateauing), PBS was pumped along the sensor chip, and we observed antibody dissociation from the surface. Using the obtained sorption-desorption sensogram shown below (Fig. 7a), the equilibrium dissociation constant was calculated to be (1.3 ± 0.1) × 10^−8^ M. This indicated insignificantly higher affinity of interaction of anti-thyroxine antibody with L-thyroxine than that with T4-bt conjugate.

To verify the specificity of interactions between thyroxine and monoclonal anti-thyroxine antibody, we conducted two independent control experiments. The solution of 100 μg/ml antibody against thyroxine and 100 µg/ml of free L-thyroxine in PBS was passed along the sensor chip with L-thyroxine immobilized on its surface. According to the obtained sensogram, antibody did not associate with surface (Fig. 7b). When passing along the sensor chip surface of the solution of 100 μg/ml nonspecific antibody (mouse antibody against human IgG), no binding was observed (Fig. 7с). The relation between free thyroxine and biotinylated thyroxine are discussed in [1].

## 7 Experimental design, materials, and methods

### 7.1 Materials

The following reagents were used in the experiments: triiodothyronine (T3), monoclonal anti-T4 antibody, biotinylated T4, thyroid-stimulating hormone (TSH), and calibration samples of human serum of different concentrations of fT4 were provided by Russian Cardiology Research and Production Complex of Russian Ministry of Health (Russia); N-(3-dimethylaminopropyl)- N-ethylcarbodiimide hydrochloride (EDC), 2-(N-morpholino) ethanesulfonic acid, human serum albumin (HSA), biotin, human immunoglobulin G and biotin N-hydroxysuccinimide were purchased from Sigma-Aldrich (Germany); nitrocellulose membrane UniSart CN140 (260 μm thick and 100 μm backing) was donated by Sartorius AG (Germany); absorbent sinks/wicking pads were purchased from Ahlstrom CytoSep (Finland); backing cards were obtained from Lohmann (USA); anti-folic acid antibody used as non-specific antibody (FA1 clone) was purchased from the Russian Research Center for Molecular Diagnostics and Therapy (Russia); streptavidin was purchased from Thermo Fisher Scientific (USA); 198 nm carboxyl-modified (COOH^−^) superparamagnetic Bio-Estapor Microspheres M1-020/50 were purchased from Estapor–Merck Millipore (Germany); anti-mouse antibody labelled with horseradish peroxidase (HRP) were purchased from Imtek (Russia). All other chemicals were of analytical grade.

### 7.2 Method of magnetic particle quantification (MPQ)

The magnetic nanolabels were counted by the original MPQ-readers [2], [3]. The MPQ readers are based on the method of non-linear magnetization of superparamagnetic nanoparticles subjected to an alternating magnetic field excited at two frequencies and registration of the magnetic signal at the linear combination of the frequencies. Such frequency mixing permits reliable registration of fine magnetic phenomena in the presence of strong noise [4] in different samples regardless of their optical transparency. The parameters of used MPQ-readers were reported earlier in the works devoted to ultrasensitive detection of disk-shaped nanoparticles [5] and multiplex detection of several biomarkers [6], [7].

### 7.3 Characterization of binding kinetics of immunoreagents

We used label-free biosensors based on the spectral-phase interferometry (SPI) [8] and spectral-correlation interferometry (SCI) [9], [10] to evaluate the kinetic binding and performance of immunoreagents. The biosensors provide real-time monitoring of changes in thickness of a biomolecular layer bound on the sensor chip surface, averaged over the sensing area [11], [12]. The kinetic and equilibrium dissociation constants for interactions are determined using the models developed earlier [13], [14]. Other procedures and protocols are described in details in Ref. [1].
